# Cytotoxicity of Air Pollutant 9,10-Phenanthrenequinone: Role of Reactive Oxygen Species and Redox Signaling

**DOI:** 10.1155/2018/9523968

**Published:** 2018-06-10

**Authors:** Manli Yang, Hassan Ahmed, Weidong Wu, Bijie Jiang, Zhenquan Jia

**Affiliations:** ^1^Department of Environmental and Occupational Health, School of Public Health, Xinxiang Medical University, Xinxiang, Henan Province 453003, China; ^2^Department of Biology, University of North Carolina at Greensboro, Greensboro, NC, USA

## Abstract

Atmospheric pollution has been a principal topic recently in the scientific and political community due to its role and impact on human and ecological health. 9,10-phenanthrenequinone (9,10-PQ) is a quinone molecule found in air pollution abundantly in the diesel exhaust particles (DEP). This compound has studied extensively and has been shown to develop cytotoxic effects both in vitro and in vivo. 9, 10-PQ has been proposed to play a critical role in the development of cytotoxicity via generation of reactive oxygen species (ROS) through redox cycling. This compound also reduces expression of glutathione (GSH), which is critical in Phase II detoxification reactions. Understanding the underlying cellular mechanisms involved in cytotoxicity can allow for the development of therapeutics designed to target specific molecules significantly involved in the 9,10-PQ-induced ROS toxicity. This review highlights the developments in the understanding of the cytotoxic effects of 9, 10-PQ with special emphasis on the possible mechanisms involved.

## 1. Introduction

Atmospheric pollution has been an important topic in the scientific and political community due to its impact on human and ecological health [1]. With industry development, the increase in city heating, and the number of automobiles, there was a severe increase in the concentration of air pollutants[2]. After the Great Smog event in London in 1952[3], the scientific community and politic governments are paying more attention to air pollution on human health [1]. Over the Great Smog event days, mortality in the general population was more than threefold times than expected, leading to thousands of people death[2]. Since this historical air pollution episode, a large number of studies have demonstrated the association between air pollution and human disease including respiratory and lung diseases such as chronic obstructive pulmonary disease, asthma attacks, pulmonary cancer, leukemia, birth defects and immune system defects, cardiovascular problems, heart disease and stroke, neurobehavioral disorders, and liver and other types of cancer [4–18]. Diesel combustion to generate power has existed for well over a century and is used in heavy machinery such as large trucks and oil tankers [19–22]. The mechanism by which diesel exhaust impacts human health is being studied extensively [23–31]. One of the most significant and detrimental impacts diesel combustion is atmospheric pollution.

Diesel particles can either aggregate or remain as individual particles when released into the atmosphere. Upon inhalation, these fine particles can penetrate through the lungs causing many diseases and disorders to develop over time. DEP have been shown to play an important role in the generation of pulmonary-related diseases including bronchitis, asthma, carcinogenesis in the lungs, and other allergic disorders [23–32]. DEPs are composed of complex mixtures of particulates, including elemental carbon, polycyclic aromatic hydrocarbons (PAHs), nitro-PAHs, quinones, heterocyclics, aldehydes, and other hydrocarbons[33–36].

**Figure 1:**
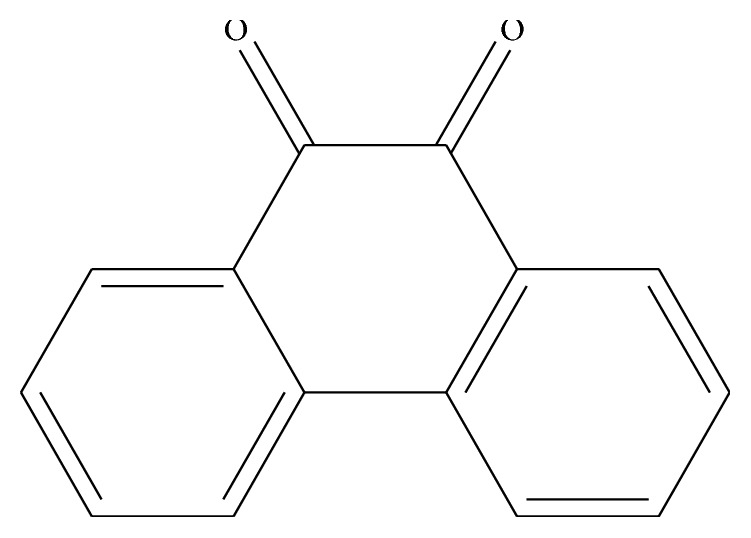
9-10-phenanthrenequinone structure.

One major particle found in diesel exhaust is 9-10-phenanthrenequinone (9,10-PQ, see its structure in Figure 1), a type of quinone. Quinones are electrophilic compounds derived from aromatic organic molecules through oxidation. These molecules have two ketone groups and, thus, are biologically active. Understanding the toxic role of quinones is of great interest as this allows for the development of novel therapeutics designed to specifically target downstream mediators. Exposure to 9,10-PQ containing particulate matter (PM) has been associated with the development of many disease processes including lung cancer, asthma, and allergic inflammation [37–52].

## 2. 9,10-Phenanthrenequinone Is One of the Major Components of Particulate Matter Present in the Environment

As one of the most abundant quinones found in DEPs [43], when inhaled, 9,10-PQ can be generated from phenanthrene by P-450s as below pathway [37]. Phenanthrene is one of major PAHs present in the air pollution [37–52].

In addition to phenanthrene photooxidation, 9,10-PQ could be also directly emitted to the environment from auto exhaust sources such as diesel exhaust particles. The levels of airborne DEP have increased dramatically over the last few decades due to the increase in the use of diesel-based engines. Compared to gasoline-based engines, diesel-based engines can provide higher fuel efficiency and lower carbon dioxide emissions; they also emit about 30–100 times more particulate matter into the atmosphere than gasoline-based engines [53]. It has been estimated that DEP constitute as much as 40% of the respirable particulate matter in a city where the daily human intake has been estimated to be as much as 300 *μ*g m^−3^ of particulate matter [54]

A few studies determined the 9,10-PQ concentration in the airborne particulates [55, 56]. Various PAH-quinones including 1-acenaphthene, 9-fluorenone, 11H-benzo[a]fluoren-11-one, 7H-benzo[c]fluoren-7-one, 11H-benzo[b]fluoren-11-one, benzanthrone, and 6H-benzo[cd]pyrene-6-one, 1,4-naphthoquinone, phenanthrenequinone, 5,12-naphthacenequinone, and benzo[a]pyrene-6,12-dione have been detected at less than 1 ppm in the atmospheric particles in Boston, MA [55, 56]. The four quinones, 1,2-naphthoquinone (1,2-NQ), 1,4-naphthoquinone (1,4-NQ), 9,10-phenanthraquinone (9,10-PQ), and 9,10-anthraquinone (9,10-AQ), have been detected in Standard Reference Material (SRM) 1649a and in ambient air samples of PM_2.5_ from several rural and urban areas in Central Los Angeles [55, 56]. The mean concentrations of these four quinones are 9–40.4 *μ*g/g in the DEP and 5–730 pg/m^3^ in the PM_2.5_ samples [55, 56]. Among these four detected quinones, 9,10-PQ has been found to have higher concentration [55, 56]. Kishikawa et al. employed a high-performance liquid chromatography with fluorescence detection method for detecting 9,10-PQ in the sample of PM collected at an urban site in Nagasaki city, Japan, weekly over a year from July 1997 to June 1998 [57]. This method is highly sensitive and allows detecting 9,10-PQ at very low concentrations of airborne particulates [57]. The average concentration of 9,10-PQ in airborne particulates was reported to be 0.287+/-0.128 ppm. Interesting, 9,10-PQ concentrations were higher in winter than in summer. Also, 9,10-PQ concentrations were higher during weekdays than at weekends [57].

9,10-PQ concentration in the PM has been investigated in a few areas during the different season. The phenanthrenequinone mean concentrations in Fresno, CA in winter 2004-2005, and spring 2005 are 1.1±0.77ng·m^−3^ and 0.31±0.13ng·m^−3^, respectively [58]. However, phenanthrene average concentrations in urban ambient Southern California are 50.34 ng·m^−3^ measured over the period of September 8-9, 1993[59]. 9,10-PQ in Central Los Angeles was found to be 24.19*μ*g/g in DEP[60]. So, the atmospheric PM2.5 and DEP consist of significant levels of 9,10-PQ [61]. High levels of 9,10-PQ were found in samples collected in San Dimas and in Riverside compared to rural Atascadero due to lower automotive exhaust emissions in rural Atascadero than that in San Dimas and in Riverside [60].

## 3. Redox Cycling of 9,10-PQ in the Generation of Reactive Oxygen Species (ROS)

**Figure 2:**
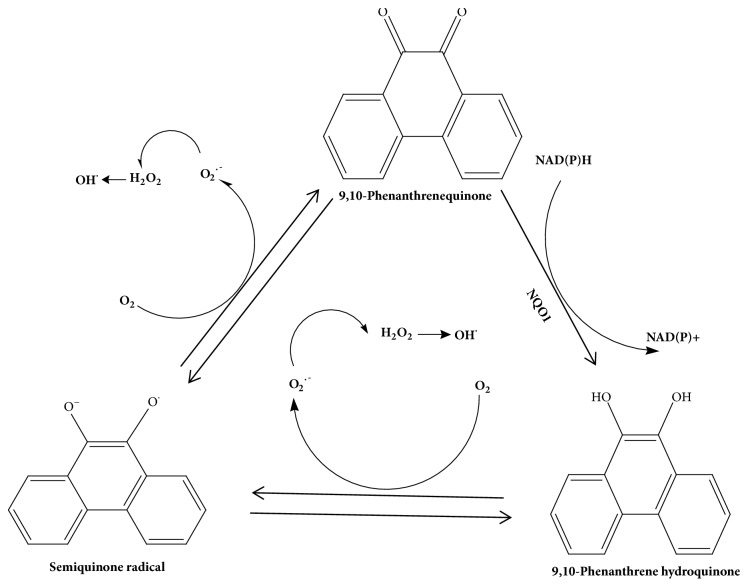
Redox cycling of 9,10-phenanthrenequinone (9,10-PQ) for production of reactive oxygen species (ROS). First, 9,10-PQ quinone undergoes 1 electron reduction to produce semiquinone radical. Semiquinone is unstable and is very reactive to oxygen. It reoxidizes back to quinone and releases superoxide radical. Superoxide molecule is then reduced to hydrogen peroxide by the superoxide dismutase. Hydroxyl radicals are formed when hydrogen peroxide reacts with metals such as ferrous ions via Fenton reaction. Second, semiquinone can also undergo further 1 electron reduction to form 9,10-phenanthrene hydroquinone which can further redox cycles back to semiquinone by losing an electron and yielding superoxide radical. Lastly, 2-electron reduction of 9,10-PQ quinone by NADPH-quinone oxidoreductase-1 (NQO1) will directly form 9,10-phenanthrene hydroquinone. Thus, redox cycle of 9,10-PQ will generate a large amount of ROS.

As a redox-active quinone in diesel exhausts, 9,10-PQ has been shown to generate ROS (Figure 2) via its redox cycling resulting in its toxicity [37–52, 57, 62, 63, 67, 71–88] in vitro and in vivo. 9,10-PQ can accept an electron to produce the semiquinone form of 9,10-PQ [70]. Incomplete reduction of molecular oxygen can cause the redox cycling of 9,10-PQ as the semiquinone form is oxidized back to the original quinone form [70]. During this process, the molecular oxygen becomes incompletely reduced and becomes a superoxide anion radical (O_2_^•-^) [89]. Superoxide radicals, considered as primary ROS, then undergo further metabolism and interact with other molecules to generate secondary ROS such as hydrogen peroxide (H_2_O_2_) and highly reactive hydroxyl radicals (^•^OH) [90]. ROS are often characterized by their capability to bring an incredible amount of both discriminant and indiscriminate damage to biomolecules including protein, lipids, and nucleic acids. In this context, there are two reduced 9,10-PQ species of biological importance, its semiquinone radical (9,10-phenanthraquinone; PQU−) and its hydroquinone PQH2. The semiquinone form of 9,10-PQ can trigger redox cycling by transferring electrons from a source such as nicotinamide adenine dinucleotide phosphate (NADPH) to oxygen to generate ROS. The semiquinone form of 9,10-PQ was found to be generated via the enzymatic reaction or nonenzymatic reaction. The enzymatic reaction was catalyzed by flavoenzymes such as NADPH-cytochrome P450 reductase or neuronal nitric oxide synthase and a nonenzymatic reaction was targeted with proximal protein thiols.

H_2_O_2_ levels within cells can be used to develop an understanding of how oxidative stress and the generation of ROS is involved in cell-induced apoptosis [91–96]. Treatment of human T lymphoblast: acute lymphoblastic leukemia (MOLT-4) cells with DCFH-DA as a fluorogenic probe can detect the levels of hydrogen peroxide within the cell. DCFH-DA is converted to DCFH which additionally is converted to 2', 7' –dichlorofluorescein (DCF) by H_2_O_2_. If H_2_O_2_ is present, this conversion will occur, and DCF levels can be measured by fluorescence to determine the levels of hydrogen peroxide within the cell. Cells treated with 9,10-PQ were shown to have a significant increase in H_2_O_2_ levels than control cells [50]. An interesting approach and technique that can be used to further understand and thereby strengthen the idea of the presence of ROS within the cells are to treat cells with 9,10-PQ as well as ROS scavengers such as polyethylene glycol catalase (PEG-cat) [50]. Upon treatment with PEG-cat and 9,10-PQ, the levels of hydrogen peroxide returned to basal control levels, indicating that 9,10-PQ indeed is involved in the generation of ROS. In addition, pretreatment of cells with PEG-cat and subsequent treatment with 9,10-PQ resulted in increased cellular viability. This further explains the role of 9,10-PQ in the generation of ROS and how ROS scavengers are involved in reducing the detrimental effects posed to the cells upon exposure to 9,10-PQ.

## 4. Role of L-Xylulose Reductase (XR) in 9,10-PQ-Induced Superoxide Production

XR is involved in the reduction of 9,10-PQ at a higher rate than its substrates diacetyl and L-xylulose [50, 97]. This enzyme was thought to be involved in cellular detoxification by reducing *α*-dicarbonyl compounds, thus reducing reactive carbonyl compounds. In contrast to what was expected, upon treatment of bovine aortic endothelial cells (BAEC) containing a sixfold overexpression of the XR enzyme with 9,10-PQ, cell viability drastically decreased and significantly enhanced cytotoxicity when compared to wild-type cells treated with only 9,10-PQ [50]. Selective inhibition of XR by 4-methyl-[1,2,3]-thiadiazole-5-carboxylic acid benzyloxy-amide (MTB) eliminated this enhanced cytotoxicity indicating that XR plays a crucial role in decreasing cellular viability when exposed to 9,10-PQ [50]. Furthermore, when XR transformed cells were pretreated with PEG-cat, GSH ester, and N-acetyl-L-cysteine (NAC), the XR induced enhanced cytotoxicity was significantly reduced [50].

Cytochrome C is an enzyme that can be used to measure the production of superoxide anion, a very powerful radical generated in the ROS pathway. As superoxide anion levels increase the reduction rate of cytochrome C also increases. In a reaction mixture containing cytochrome C, XR, and 9,10-PQ, decrease in levels of cytochrome C significantly increased compared to the control mixture lacking 9,10-PQ indicating the generation of the superoxide anion. Also, when the reaction mixture was treated with CuZn-SOD, a compound involved in the conversion of superoxide anion into oxygen and H_2_O_2_, cytochrome C reduction levels decreased, further explaining the role of XR in enhancing 9,10-PQ induced cytotoxicity through the generation of superoxide anion [98].

After establishing the relationship between XR and cytotoxicity, it is crucial to understand how the induction levels of XR change upon exposure to 9,10-PQ. When MOLT-4 cells were treated with 1 uM 9,10-PQ, XR mRNA levels were increased 2 hours after treatment, indicating that XR may be an oxidant-inducible enzyme. Cells treated with low concentrations of 9,10-PQ resulted in a greater expression of XR mRNA, as opposed to cells treated with higher concentrations of 9,10-PQ. A probable explanation for this phenomenon can be obtained from the DNA fragmentation results. As the cells were treated with higher concentrations of 9,10-PQ increase in DNA fragmentation was observed. This increase in DNA fragmentation can be a cause of reduced expression of XR mRNA levels at higher concentrations of 9,10-PQ. Furthermore, to strengthen the idea of 9,10-PQ induced upregulation of XR mRNA expression, cells exposed to 9,10-PQ were pretreated with radical scavengers PEG-cat and NAC to understand how XR mRNA levels may change [98]. XR mRNA levels were indeed reduced upon pretreatment of cells with radical scavengers; thus, it can be concluded that enhanced expressions of XR mRNA in the presence of 9,10-PQ can be mitigated upon pretreatment with PEG-cat and NAC. Upregulation of XR mRNA was not only observed in cells treated with 9,10-PQ, cells treated with glucose oxidase (GO) and glucose, a hydrogen peroxide generating system, also increased the expression of XR mRNA levels indicating the strong relationship between ROS generation and upregulation of XR. Knowing this relationship between ROS generation and enhanced XR levels, it can be predicted that treatment of cells with XR inhibitory factors can reduce ROS generation levels as well as increasing cell viability levels when compared to cells treated with only 9,10-PQ without XR inhibitors [98]. Indeed, upon treatment of cells with MTB, an inhibitor of XR, cytochrome C reduction significantly decreased and cellular viability significantly increased. These results further strengthen the important role that XR plays in the molecular pathway of 9,10-PQ induced cellular toxicity.

## 5. 9,10-PQ and Cellular Toxicity

Many studies demonstrated that PQ can cause toxicity in vivo and in vitro biological system via the redox cycling for the generation of ROS [42, 46, 47, 50, 72, 78, 82]. Excessive ROS is known to cause DNA damage resulting in gene mutation [99–103]. 9,10-PQ has been shown to exert toxic effects in human skin cell lines with low NQO1 activity [45]. Nitric oxide (NO) is widely known to suppress cytotoxic effects of oxidative damage. Pretreatment of endothelial cells resulted in decreased toxicity of 9,10-PQ and conversely, depletion of NO resulted in a greater level of toxicity, signifying the role of NO in cellular detoxification [48]. Interestingly, antitumor studies have been performed using analogs of 9,10-PQ due to their role in apoptosis. In HCT-116 colon tumor cells and HL-60 promyelocytic leukemia cells, 9,10-PQ analogs were successful in inducing apoptosis [43]. In the trophoblast cell line, JEG-3, 9,10-PQ exerted greater amounts of cytotoxicity and increased levels of redox cycling were measured when coupled with copper [67]. 9,10-PQ has been shown to cause iron-mediated cellular damage in human pulmonary epithelial cells. 9,10-PQ induced apoptosis and downregulated the effects of CuZn-SOD, which converts superoxide anion into oxygen or H_2_O_2_ [52]. PQH_2_, a hydroquinone of 9,10-PQ, has shown to play a significant role in generating oxidative stress, protein damage, and cellular toxicity [70]. Exposure to 9,10-PQ can be examined in urine specimen by testing for the presence of 9,10-PQHG, a major metabolite generated during the breakdown of 9,10-PQ. This metabolite of 9,10-PQ was detected in rats injected with 9,10-PQ as well as humans exposed to diesel exhaust particles [63].

One of the most crucial experiments highlighting the molecular mechanisms of 9,10-PQ induced cellular toxicity includes a study performed by Matsunaga et al. in 2007. This study evaluated the effect of 9,10-PQ on various cellular mechanisms such as apoptosis, generation of ROS, and the role of the cytoprotective enzyme XR in the apoptotic signaling and ROS generating pathway in BAECs [50].

## 6. The Role of 9,10-PQ in Cellular Viability and DNA Damage

In a study carried out by Matsunaga et al., it has been shown that 9,10-PQ induces apoptosis in MOLT-4 cells similar to other quinones such as 1,2-naphthoquinone and 1,4-naphthoquinone. MOLT-4 cells are human T lymphoma cells, obtained from cancerous T cell leukemia tissue [50]. The use of cancerous tissue in the study of apoptosis can be very useful in the development of not only anticytotoxic treatments but also antitumor treatments. One of the most important aspects of understanding toxicity is measuring the effects of a compound on cellular toxicity. 9,10-PQ was shown to decrease the viability of MOLT-4 cells as early as 8 hours after treatment. Cells were also treated with phenanthrene to measure cellular toxicity. The purpose of measuring toxicity upon treatment with phenanthrene was to explain the significance the quinone group has on biological activity, as the structure of phenanthrene is similar to 9,10- PQ except it lacks the quinone group. Phenanthrene did not have significant effects on the viability of the cells. Dose-dependent response to cellular toxicity was also measured in this experiment using varying concentrations of 9,10-PQ, 1,2-NQ, 1,4-NQ, acenaphthenequinone, and phenanthrene. The results suggest that all the quinones except acenaphthenequinone contributed significantly to cell viability upon treatment with 1 uM of the quinone [50].

DNA fragmentation occurs when the integrity of the nuclear membrane is compromised by exposure to xenobiotic compounds, which in turn causes morphological changes of the nucleus and DNA. These changes are pivotal features and steps in cells undergoing apoptosis or programmed cell death. Upon treatment of MOLT-4 cells with 9,10-PQ, DNA was extracted and analyzed via gel electrophoresis [50]. A clear and transparent increase in fragmentation of the DNA was observed in cells treated with 0.05 to 5 uM of 9,10-PQ. Treatment with 5 uM of phenanthrene did not have any effect on the fragmentation of DNA, further strengthening the idea that the quinone portion of 9,10-PQ is a vital component of the molecule necessary in the exertion of nuclear toxicity in cells. A separate study performed by Rodriguez et al. on* Saccharomyces cerevisiae* analyzed the role of 9,10-PQ in DNA damage, specifically examining deletions and point mutations in DNA [49]. The results specify that 9,10-PQ plays a role in DNA damage only when treated to cells in the presence of oxygen. No changes in DNA abnormality were observed within cells treated with 9,10-PQ in anaerobic conditions in yeast cells indicating oxygen is required by cells undergoing 9,10-PQ induced DNA damage. Also, an important protein involved in the repair of DNA is Poly (ADP-ribose) polymerase (PARP) [50]. The levels of activated PARP can give an indication as to the amount of stress, specifically DNA damage, a cell is experiencing [104–107]. PARP repairs DNA and increase in DNA damage triggers an increase in PARP activity. 9,10-PQ has been shown to increase the levels of activated PARP inside cells, further strengthening the role of 9,10-PQ in DNA damage. Nucleic morphology can provide imperative clues in understanding the methodology by which cells are undergoing death.

The two main types of cell death are apoptosis and necrosis [108–111]; noteworthy, molecular and morphological differences are observed in each type of cell death. Apoptosis is referred to as programmed cell death involving well-organized sequence of morphological events [108–111]. Nuclear and cytoplasmic membranes of cells undergoing apoptosis shrink and condense which results in the collapse of the cytoskeleton causing the formation of blebs by the cell membrane. These cells subsequently undergo degradation of the genetic and protein material as the nucleus is condensed and fractured. The cellular debris or blebs are eventually phagocytosed by neighboring cells or macrophages [108–111]. On the contrary, necrosis is referred to as accidental or unscheduled cell death. Necrotic cells swell and distend which drastically alters their structure due to an inability to maintain membrane integrity. This in turn ultimately results in the damage and destruction of the organelles, and the interior composition of the cells leak out. Nucleic morphology of cells treated with 9,10-PQ displayed the formation of condensed and fragmented nuclei. These changes explain the noteworthy role of 9,10-PQ in the induction of apoptosis [50].

## 7. The Role of 9,10-PQ on Mitochondria Dysfunction and Caspase Activation

The mitochondria play an essential role in the steps leading towards apoptosis; thus, studying this organelle is vital in understanding how xenobiotic factors impair the activity of cells through this organelle [112–114]. Three key proteins located on the inner mitochondrial membrane include the apoptotic Bax and the antiapoptotic Bcl-2 and Bcl-XL play a crucial role in regulating the permeability of the membrane [115–119]. Upregulating the activity of Bax and downregulating the activity of Bcl-2 and Bcl-XL result in the cell progressing towards apoptosis. Other proteins involved in the apoptotic pathway include cytochrome C and cysteine-aspartic proteases (caspase) 3, caspase 8, and caspase 9. Caspases are a family of cysteine-dependent aspartate-directed proteases which play a critical role in the transduction of apoptotic signals [120, 121]. Cytochrome C is a protein normally localized in the mitochondria, but upon receiving intrinsic apoptotic signals, cytochrome C translocates to the cytosol where it interacts with apoptosis-activating factor 1 (Apaf-1) and caspase 9 which play a crucial role in the activation caspase 3 [122–124]. Activation of caspase 3 results in the apoptosis of the cell. Treatment of MOLT-4 cells with 9,10-PQ resulted in the decrease of Bcl-2 and Bcl-XL proteins and an increase in the Bax protein as well as an upregulation of cytosolic cytochrome C levels indicating mitochondrial damage and cell progression towards apoptosis [50]. Also, the membrane potential of the mitochondria was notably reduced. Increase in the activity of caspases 3, 8, and 9 was also observed. Time-dependent experiments were performed to understand the precise time caspases 3, 8, and 9 were activated. To trigger apoptosis caspase 3 should be activated after caspases 8 and 9. Based on the analysis caspase 3 activity increased by approximately 8-fold 8 hours after treatment with 9,10-PQ, whereas caspases 8 and 9 activity was increased by approximately 4-fold, the highest level, after 6 hours [50]. From this data, a possible mechanism involved in triggering apoptosis includes activation of caspase 8 upon exposure to 9,10-PQ. Activated caspase 8 eventually downregulates antiapoptotic Bcl-2 and Bcl-XL proteins and upregulation of the proapoptotic Bax protein. Increase in Bax activity causes Bax to translocate into the mitochondria causing a reduction in the mitochondrial membrane potential and increase in the permeability of the mitochondria, which ultimately results in the release of cytochrome C into the cytoplasm of the cell, triggering caspase activated apoptosis [50].

## 8. The Role of 9,10-PQ in Glutathione and Cellular Detoxification

A major component of cellular detoxification involves Phase II reactions which are responsible for increasing the hydrophilicity of the xenobiotic compounds to aid in the excretion process [125]. Phase II reactions are often referred to as conjugation reactions because of these reactions couple hydrophilic groups to xenobiotic compounds. One of the most studied Phase II coupling reactions involves glutathione conjugation. GSH is a tripeptide which acts as a critical component in the detoxification process of cells undergoing oxidative stress [126–128]. This thiol compound containing a reactive sulfhydryl group is a very powerful and important antioxidant. The enzyme responsible for this crucial reaction is referred to as glutathione transferase (GST), which adds glutathione to the parent xenobiotic compound either by direct addition or by replacement of an electrophilic substituent. Although GSH acts as a cofactor for GST, it can independently act as a free radical scavenger [126–128]. To understand the role of 9,10-PQ in GSH levels, it can be predicted that GSH levels will be low upon exposure to 9,10-PQ. Upon treatment of MOLT-4 cells with 9,10-PQ in a time-dependent manner GSH levels were significantly decreased inside the cells to 40 percent of the original concentration after 4 hours and after 24 hours GSH levels were approximately 10 percent of the original concentration [50]. GSH levels drop significantly when cells are undergoing oxidative stress as this mechanism is very vital in the antioxidant detoxification process. To further prove and confirm the depletion of GSH was indeed caused by 9,10-PQ induced oxidative stress and apoptosis, MOLT-4 cells were pretreated with varying concentrations of cell-permeable GSH monoethyl ester (NAC) to mimic the effects of GSH before being exposed to 5 uM concentrations of 9,10-PQ [50]. Pretreatment of cells with 1 and 2 mM NAC significantly increased cell viability, thereby protecting the cells against 9,10-PQ induced cell death. NAC also protected the cells against DNA fragmentation, although GSH was not directly involved in DNA repair. These results suggest the relative significance and importance of antioxidant compounds, namely, GSH, in the cellular detoxification process as well as maintaining the viability of cells.

## 9. The Role of 9,10-PQ in Cardiovascular Disease

Common types of the cardiovascular disease mainly include hypertension, coronary heart disease (CHD), stroke, rheumatic heart disease, congenital heart disease, and congestive heart failure (CHF)[129]. Despite great progress has been made in the management of CVDs, CVD do harm to human health because of the high morbidity and mortality. It has been shown that about 63% of premature deaths in adults (aged 15–69 years) and three-out-of-four of all adult deaths are mainly attributed to CVD [130]. It is shocking that, by 2030, over 23.3 million people will die annually from CVD[131].A large number of researches have shown that long-term exposure to fine particulate matter, known as PM2.5, can impact heart disease [132–144]. Both acute and chronic PM exposures can significantly increase the cardiovascular morbidity and mortality [133–144].

Previous studies demonstrated that 9,10-PQ can elicit adverse cardiovascular effects such as alterations in vascular reactivity [48, 72, 82, 145–147] and aortic ring relaxation [53]. Exposure to diesel exhaust containing 9,10-PQ was found to diminish humans forearm blood flow through endothelium-dependent and -independent mechanisms [148]. In the animal experiment, it was found that gaseous components of diesel exhaust can enhance vasoconstriction and suppress vasodilation in septal coronary arteries of mice [146]. An animal study examined the effects of phenanthraquinone on eNOS activity, endothelium-dependent relaxation, and blood pressure [53]. Exposure of rats to phenanthraquinone (0.36 mmol/kg) via an intraperitoneal administration was shown to result in the significant elevation of blood pressure [53]. Administration of phenanthraquinone to rats also decreased the level of NO2^−^/NO3 in plasma. NO2^−^/NO3 has been considered to be an excellent indicator of NO production in vivo [53]. NO production was also suppressed by phenanthraquinone in membrane fraction of BAECs with a concentration-dependent manner by measuring citrulline formation from L-arginine [149]. Thus, these in vitro and in vivo studies demonstrated that 9,10-PQ can cause an impairment of endothelium-dependent vasorelaxation and are associated with cardiopulmonary-related diseases and mortality (Table 1) [48, 72, 82, 147].

## 10. Conclusion

9,10-PQ is a quinone molecule found in air pollution abundantly in the DEP. This compound has been studied extensively and has been shown to develop cytotoxic effects both in vitro and in vivo. Cytotoxicity generated by 9,10-PQ has been proposed to be mediated by its cycling for ROS generation. Exposure to 9,10-PQ can activate apoptotic factors such as caspases. Understanding the molecular mechanisms involved in the ROS pathway can allow for the development of novel and effective treatment of 9,10-PQ-mediated toxicity.

## Figures and Tables

**Table 1:** Major 9,10-phenanthraquinone studies and mechanisms of action.

Source	Concentrations/Doses of 9,10-PQ	Models (*in vitro/in vivo*)	Results/ mechanisms of action
Muraki et al. (2017) [62]	0.1–30 *μ*M	Human embryonic kidney (HEK) cells and alveolar A549 cells	The results showed that 9,10‐PQ activated human TRPA1 via critical cysteine residues at 621 and 665 in the N‐terminus of the channel.

Asahi et al. (2014) [63]	50 mg/kg for male rat	Urine samples from Male rats and human	In rat urine samples following 9,10-PQ exposure, the monoglucuronide of 9,10-dihydroxyphenanthrene (9,10-PQHG) was found to be a major metabolite. 9,10-PQHG also present in human urine.

Hatae et al. (2013) [43]	10*μ*M	HCT-116 colon tumor cells and HL-60 promyelocytic leukemia cells	Effects of orthoquinone moiety in 9,10-phenanthrenequinone on apoptosis were studied in HCT-116 and HL-60 cells. Results showed that the loss of the cis-orthoquinone unit in 9,10-PQ abrogated its ability to induce apoptosis in HCT-116 and HL-60 cells suggesting that cis-orthoquinine is an essential unit for 9,10-PQ to induce tumor cells apoptosis

Koizumi et al. (2013) [64]	10*μ*M or 100*μ*M	In Vitro Enzyme reaction	Redox cycling of 9,10-PQ in the presence of dihydrolipoic acid can cause oxidative damage of Cu, ZnSOD, which are associated with decreased enzyme activity.

Toyooka et al. (2012) [45]	0-25*μ*M	A549 and MCF7 cell lines	In low NQO1-expressing cells, N-acetyl-L-cysteine (NAC) significantly enhanced 9,10-PQ-mediated cytotoxicity and the formation of DNA double-strand breaks with phosphorylation of histone H2AX. In contrast, 9,10-PQ-mediated cytotoxicity and genotoxicity were suppressed in presence of NAC in high NQO1-expressing human adenocarcinoma cell lines, A549 and MCF7. The results suggested that dual effects of NAC on 9,10-PQ-mediated cytotoxicity and genotoxicity are dependent on the NQO1 activity

Sugimoto et al. (2005) [65]	5-30*μ*M	A549 human pulmonary epithelial cells	9,10-PQ induced apoptosis in A549 cells and the LC50 is ~7*μ*M. Furthermore, 9,10-PQ induced the formation of hydroxyl radical in the presence of iron, NADPH, and P450 reductase in A549 cells. Chelating iron provided protection against the PQ-induced cytotoxicity. Treatment of A549 cells with PQ caused a decrease in levels of Cu,Zn–superoxide dismutase (Cu,Zn–SOD) and heme oxygenase-1 (HO-1) indicating that 9,10-PQ exerted oxidative stress in human A549 cells via iron-mediated oxidative damage with down-regulation of Cu, Zn-SOD.

Rodriguez et al. (2008) [49]	Aerobic conditions: 2.5-20*μ*M; Anaerobic conditions: 20-50*μ*M	yeast Saccharomyces cerevisiae	9,10 PQ can inhibit yeast S. cerevisiae growth under both aerobic and anaerobic conditions. However, 9,10 PQ induced DNA deletions and point mutations only in the presence of oxygen, not anaerobic conditions.

Taguchi et al. (2007) [66]	1-20*μ*M	A549 human pulmonary epithelial cells	The results showed that PQH_2_ is the product of an NADPH-dependent two-electron reduction of 9,10-PQ. NADPH-dependent enzymes such as the AKR1C isozyme can mediate the two-electron reduction of 9,10-PQ to PQH_2_ resulting in redox cycling with 9,10-PQ, which is associated with oxidative protein damage

Peters et al. (2007) [67]	0-100*μ*M	JEG-3 trophoblast cell line	9,10-PQ induced a concentration-dependent decline in Alamar Blue (AB), suggesting an impairment to energy metabolism. 9,10-PQ-mediated cytotoxicity was dramatically enhanced by the addition of copper.

Milko et al. (2009) [68]	TSQ Classic mass spectrometer with a QOQ configuration chemical reaction, 9,10-PQ as a ligand	Gas-phase model complexes [(PQ)FeCl(CH(3)O)](+), [(phen)FeCl(CH(3)O)](+), and [(PQ)(phen)FeCl(CH(3)O)](+)	The results showed that there is an interaction between iron(III) and phenanthraquinone in the isolated complexes in the gas phase, which is is driven by the reduction of iron(III) to iron(II) and 9,10-phenanthraquinone to the semihydroquinone radical or semiquinolate.

Hiyoshi et al. (2005) [69]	0.01 nmole/animal	Male ICR mice	Administration of 9,10-PQ (2.1 ng/animal) via intratracheal route dramatically increased allergic airway inflammation in mice and allergen-specific production of IgG1 and IgE.

Matsunag et al. (2008) [50]	1-10*μ*M	human acute T-lymphoblastic leukemia MOLT-4 cells	9,10-PQ generated ROS, depleted cellular glutathione and trigged apoptosis signaling including mitochondrial membrane dysfunction and activation of caspases and poly(ADP-ribose) polymerase. 9,10-PQ-mediated ROS generation and cytotoxicity were increased in an XR-transformed cell line. Furthermore 9,10-PQ-induced cell death was partially inhibited when cells were pretreated with XR-specific inhibitors followed by 9,10-PQ exposure. These results suggest that -Xylulose reductase is critically involved in 9,10-phenanthrenequinone-induced apoptosis in human T lymphoma cells

KUMAGAI et al. (2001) [53]	In vitro (0.15-5 *μ*M), in vivo (0.36 mmole/kg body weight)	Bovine aortic endothelial cells; Wistar rats (8–10 wk old)	9,10-PQ was found to significantly inhibit nitric oxide (NO) formation in bovine aortic endothelial cells with an IC(50) value of 0.6 *μ*M. 9,10-PQ also inhibited endothelium-dependent relaxation of rat aortic rings while the endothelium-independent relaxation by nitroglycerin was not affected. Furthermore, intraperitoneal injection of 9,10-PQ (0.36 mmol/kg) to rats elevated blood pressure, plasma levels of stable NO metabolites, nitrite/nitrate compared to control group. These results suggested that 9,10-PQ is a potent inhibitory action on eNOS activity resulting in inhibition of NO-mediated vasorelaxation and elevation of blood pressure.

Taguchi et al. (2008) [70]	1–50 *μ*M	Mouse primary hepatocytes	Exposure of human pulmonary epithelial A549 cells to 9,10-PQ resulted in a time-dependent formation of 9,10-PQH2 (9,10-PQHG) and. UGT1A10 and UGT1A6 found to be particularly involved in 9,10-PQHG formation. In cell-free systems, 9,10-PQ while not 9,10-PQHG, showed a quick thiol oxidation and oxygen consumption in the presence of dithiothreitol.

Oginuma et al. (2005) [71]	10 *μ*M	Pig heart cytosol	9,10-PQ was found to be a potent inhibitor for the 4-benzoylpyridine (4-BP) reduction. and pig heart carbonyl reductase plays a critical role in superoxide generation via redox cycling of 9,10-PQ

Prisby et al. (2005)[32]	5 *μ*M for In Vitro principal nutrient artery studies	Female and male Fischer-344 (F-344) rats	9,10-PQ diminished endothelium-dependent vasodilation of PNA in 14- and 24-mo-old rats of both genders. However, there was no change in femoral PNA in 6-mo-old male rats, suggesting that ovarian hormones play important role in the vascular endothelium at this stage of development.

## Abbreviations

9,10-PQ: 9,10-phenanthrenequinone
DEP: Diesel exhaust particles
ROS: Reactive oxygen species
GSH: Glutathione
PAHs: Polycyclic aromatic hydrocarbons
PM: Particulate matter
Cu,Zn-SOD: Cu,Zn-superoxide dismutase
NQO1: Quinone oxidoreductase
XR: L-Xylulose reductase
9,10-PQH2: 9,10-dihydroxyphenanthrene
1,2-NQ: 1,2-naphthoquinone
1,4-NQ: 1,4-naphthoquinone
9,10-AQ: 9,10-anthraquinone
H_2_O_2_: Hydrogen peroxide
^•^OH: Hydroxyl radicals
NADPH: Nicotinamide adenine dinucleotide phosphate
MOLT-4 cells: Acute lymphoblastic leukemia cells
DCF: 2', 7'–dichlorofluorescein
PEG-cat: Polyethylene glycol catalase
BAEC: Bovine aortic endothelial cells
MTB: 4-methyl-[1,2,3]-thiadiazole-5-carboxylic acid benzyloxy amide
NAC: N-acetyl-L-cysteine
GO: Glucose oxidase
NO: Nitric oxide
PARP: Poly (ADP-ribose) polymerase
Caspase: Cysteine-aspartic proteases
GST: Glutathione transferase
CHD: Coronary heart disease
CHF: Congestive heart failure
CVDs: Cardiovascular diseases.
